# Experimentally Demonstrate the Spin-1 Information Entropic Inequality Based on Simulated Photonic Qutrit States

**DOI:** 10.3390/e22020219

**Published:** 2020-02-15

**Authors:** Lianzhen Cao, Xia Liu, Yang Yang, Qinwei Zhang, Jiaqiang Zhao, Huaixin Lu

**Affiliations:** Department of Physics and Optoelectronic Engineering, Weifang University, Weifang 261061, China; lianzhencao@wfu.edu.cn (L.C.); yangyang@wfu.edu.cn (Y.Y.); qwzhang@wfu.edu.cn (Q.Z.); jqzhao@wfu.edu.cn (J.Z.); huaixinlu@wfu.edu.cn (H.L.)

**Keywords:** information entropic inequality, second-order nonlinear effect, multiphoton interference enhancement effect, high dimensional quantum correlation

## Abstract

Quantum correlations of higher-dimensional systems are an important content of quantum information theory and quantum information application. The quantification of quantum correlation of high-dimensional quantum systems is crucial, but difficult. In this paper, using the second-order nonlinear optical effect and multiphoton interference enhancement effect, we experimentally implement the photonic qutrit states and demonstrate the spin-1 information entropic inequality for the first time to quantitative quantum correlation. Our work shows that information entropy is an important way to quantify quantum correlation and quantum information processing.

## 1. Introduction

Quantum correlation of high dimensional quantum systems is new, as well as important resources, which can promote the development of quantum information science and technology [1,2,3]. The quantitative complexity depends on the chosen correlation and dimension of the bipartite d-level systems. To obtain the full information of quantum correlation, it is generally necessary to measure all the results to reconstruct quantum entanglement by quantum tomography. With the dimensions *d* increasing, the results of the basis to be measured increase exponentially, which becomes very difficult to achieve experimentally. So, to experimentally quantize the quantum correlation is crucial, but difficult. In view of this, Braunstein and Caves propose famous BC theoretic-chained information entropic inequalities [4]. The inequalities are written in terms of the mean information obtained in several measurements, but not probability space, and can be applied to any pair of widely-separated systems, not just two-state systems. This means that a pair of spin-s systems can violate these information entropic inequalities for arbitrary values of s with quantum statistics.

The BC inequalities have many advantages, which Clauser–Horne–Shimony–Holt (CHSH) inequalities or other Bell type inequalities do not have. For example, they can be readily applied to quantum systems of arbitrary local dimension and general measurement operators and can also be used to evaluate the accuracy of experimental data and the value of mutual information. Another advantage of the entropic approach is that it easily adapts to situations of additional independence requirements, like the bilocality scenarios introduced by an entanglement swapping experiment [5], general correlation, and causal model scenarios [6,7]. In particular, information entropy inequality has many important applications in practical quantum computing and quantum communication. [8]. The information entropic inequality is used to solve important problems in quantum computing, such as the “database” search problem [9], marginal problem [10], and quantum contextuality [11]. The large measurable quantity-chained BC inequalities can also be used to improve the security of quantum key distribution (QED) protocols [12]. Moreover, inspired by BC inequality, the q deformed entropic functions [13,14] and the uncertainty relations have been studied busing the entropy of the quantum state [15].

All mentioned above information and entropic schemes for quantum systems open a new prospective for implementation of quantum technologies, e.g., realization of quantum memory, quantum algorithms, and many-level quantum simulation [16]. However, in order to prove the inequality and study its application experimentally, especially the higher-dimensional inequality, the quality requirement of the entangled system is very strict [17,18,19]. To our knowledge, experimental reports on quantum entanglement entropy are very few [18,19,20,21]. 

In this paper, we present the first experimental demonstration of the BC inequality for entangled spin-1 objects. Using the second-order nonlinear optical effect and multiphoton interference enhancement effect, we prepared the photonic maximally qutrit state with fidelity of 0.978. The spin-1 BC entropic inequality was demonstrated experimentally using the high-quality entanglement state. The results show that the correlation between space-like separated entangled quantum systems can be quantified easily by using quantum information entropy.

## 2. The Theory of Information Entropy Inequality 

The measurements performed on two quantum entangled systems can be used to measure the non-local correlations, in other words, the Bell inequality is violated. The most famous of these inequalities is the CHSH inequality, but only two state systems can be applied. Braunstein and Caves give an information theoretic Bell inequality, which can apply any pair of widely separated systems. Now we will briefly introduce the theoretical content of information entropy inequality. According to the mean mutual information theory, we can easily get the two inequalities: H(A|B)≤H(A)≤H(A,B). The H(A|B) is the conditional information entropy. The H(A) is the information carried by *A*. The H(A,B) is the mean information entropy. In order to study the spin-1 entropy inequality, we considered two particles, A and B, with spin = 1 and opposite directions. Each particle was sent through a Stern—Gerlach type of measurement. For particles A and B, they each had three measurable quantities, A_1_, A_2_, and A_3_, associated with A and B_1_, B_2_, and B_3_, associated with B, respectively, which interleaved in a sequence A_1_, B_3_, A_2_, B_2_, A_3_, B_1_. Each measurable quantity had three possible values and is denoted here by quantum number m=−s,−s+1,…, s−1,s.   (s=1). According to the quantum mechanical description of the probability of particles, and through the treatment of the above two inequalities, the spin-1 information entropic inequality based the conditional information entropy can be expressed as [4]:

(1) $$H\left(A_{1}|B_{1}\right)\leq H\left(A_{1}|B_{3}\right)+H\left(B_{3}|A_{2}\right)+H\left(A_{2}|B_{2}\right)+H\left(B_{2}|A_{3}\right)+H\left(A_{3}|B_{1}\right)$$

where the $H\left(A_{1}|B_{1}\right)$ is a conditional information entropy, representing the information carried by A_1_, given the value of B_1_. The other five expressions have a similar physical meaning.

In order to experimentally verify spin-1, the information entropic inequality, we need to further process Formula (1) in the statistical sense of quantum mechanics. The six observable measures, A_1_, A_2_, A_3_, B_1_, B_2_, and B_3_ mentioned earlier, are spin components specified by unit vectors $\vec{a_{1}},\vec{b_{3}},\vec{a_{2}},\vec{b_{2}},\vec{a_{3}},\vec{b_{1}}$. They are coplanar and separated by angles θ/6 (θ is the angle between $\vec{a_{1}}$ and $\vec{b_{1}}$). Formula (1) can be further expressed as:

(2) $$\Pi^{QM}\left(\theta \right)\equiv 5H^{QM}\left(\theta |5\right)-H^{QM}\left(\theta \right)$$

where the $\Pi^{QM}\left(\theta \right)$ is the information difference. According to the above-mentioned measurement method and parameter settings, Formula (1) is violated when the $\Pi^{QM}\left(\theta \right)$ is negative. The deficit information carried by the widely separated systems is expressed using the negative value. The $H^{QM}\left(\theta \right)$ stands for the quantum mechanical information, and $H^{QM}\left(A|B\right)=H^{QM}\left(B|A\right)\equiv H^{QM}\left(\theta \right)$ is given by the form:

(3) $$H^{QM}\left(\theta \right)=-1/3\sum_{m_{1},m_{2}}{\left|D_{m_{1},-m_{2}}\left(R_{n}(\theta )\right)\right|}^{2}\log{\left|D_{m_{1},-m_{2}}\left(R_{n}(\theta )\right)\right|}^{2}$$

where the $D_{m_{1}m_{2}}\left(R_{n}(\theta )\right)=\langle sm_{1}|e^{-i\theta }{}^{n\cdot S_{A}}|sm_{2}\rangle$ is a rotation matrix for the $R_{n}(\theta )$ by angle θ and can be calculated by the definition of the following equation:

(4) $${\left|D_{m_{1},-m_{2}}\left(R_{n}(\theta )\right)\right|}^{2}=\left(2s+1\right){\left|{}_{A,\vec{a}}\langle sm_{1}|{⊗}_{B,\vec{b}}\langle sm_{2}||\phi \rangle \right|}^{2}=\left(2s+1\right)P\left(a=m_{1},b=m_{2}\right)$$

From the zero totals spin state $|\Phi \rangle =1/\sqrt{3}\sum {\left(-1\right)}^{s,-m}{|s,m\rangle }_{A\vec{e}}⊗{|s,-m\rangle }_{B\vec{e}}$, the quantum statistics of spin-1 information entropic inequality can be demonstrated. For particles with spin 1, s is 1 and m is 1, −1, and 0, respectively. So the state has three distinct basis states (|−1〉,|0〉 and |1〉), and its expression is:

(5) $$|\Phi \rangle =1/\sqrt{3}(|1,1\rangle ⊗|1,-1\rangle -|1,0\rangle ⊗|1,0\rangle +|1,-1\rangle ⊗|1,1\rangle )$$

## 3. Entanglement State Experimental Implementation

State (5) can be experimentally simulated using the polarization-entangled four photon state [17,18]. It was produced by the second-order pulsed parametric down conversion field. First order nonlinear effects of barium borate (BBO) crystals produce an entangled state $1/\sqrt{2}(|H,V\rangle +|V,H\rangle )$. However, if we use the second order term of the down converted field and post selection process, the polarization entangled multiphoton mode state can be prepared, and the form is given by $1/\sqrt{3}(|2H,2V\rangle +|\mathrm{HV},\mathrm{VH}\rangle +|2V,2H\rangle )$. The H (V) is the horizontal (vertical) polarization of the photon. The state |2H,2V〉 means that if A measures two horizontal photons, then B will measure two vertical photons, and |2V,2H〉 state means the opposite. The photons sent to A (and B) have three possible polarization measurement outcomes with equal probabilities, namely |2H〉,|HV〉, and |2V〉, which can be defined as the |1〉,|0〉, and |−1〉 state, respectively. The polarization measurement is the analog of a Stern—Gerlach measurement for spin-1 particles. If we encode the |1,1〉, |1,−1〉, and |1,0〉 as |2H〉, |2V〉, and |HV〉, the $1/\sqrt{3}(|2H,2V\rangle +|\mathrm{HV},\mathrm{VH}\rangle +|2V,2H\rangle )$ is just the spin-1 entanglement State (5). 

**Figure 1:**
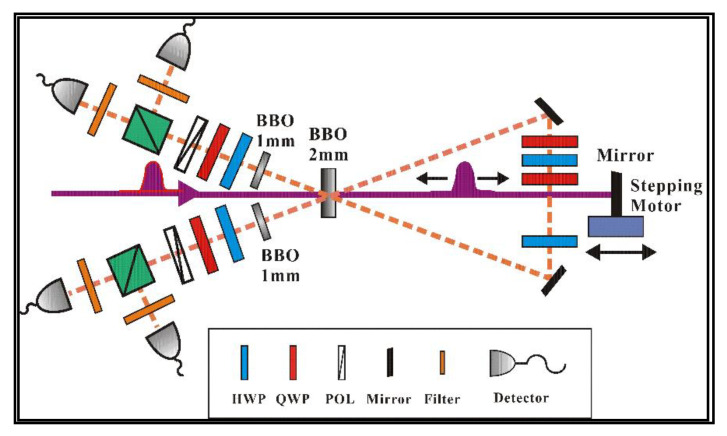
The schematic diagram of the experimental apparatus for generating the four-photon entanglement state. The four photon states are produced using the type-II noncollinear parametric down conversion process of barium borate (BBO) crystal and amplified by the optical feedback configuration structure.

Figure 1 is a schematic diagram of the experimental apparatus for generating the spin-1 entanglement state. A locked mode Ti:sapphire femtosecond laser with a repetition rate of 80 MHz and a wavelength of 390 nm was used to pump 2 mm of BBO crystal. In order to improve the brightness of the prepared entanglement state, the two-pass scheme is implemented by reflecting the down converted field into the crystal again. In order to compensate for the time and space deviation of photons in different polarization directions, the generated photons pass through the half wave plate (HWP) and 1mm BBO crystal again. This produces the entangled state we need: $1/\sqrt{3}(|2H,2V\rangle +|\mathrm{HV},\mathrm{VH}\rangle +|2V,2H\rangle )$. The analyzer contains a half wave plate (HWP), a quarter wave plate (QWP), a polarizer (POL), a polarizing beam splitter (PBS), a narrow bandwidth filter (NBF), and two single photon detectors. The |2H〉 (|2V〉) state is measured by the combination device of HWP + QWP + POL + PBS, and the POL is to make sure that only single polarized photons (horizontally or vertically) are transmitted. The |HV〉 state is detected by further setting the HWP at 0°, with respect to the horizontal polarization direction. 

## 4. Results and Discussion

**Figure 2:**
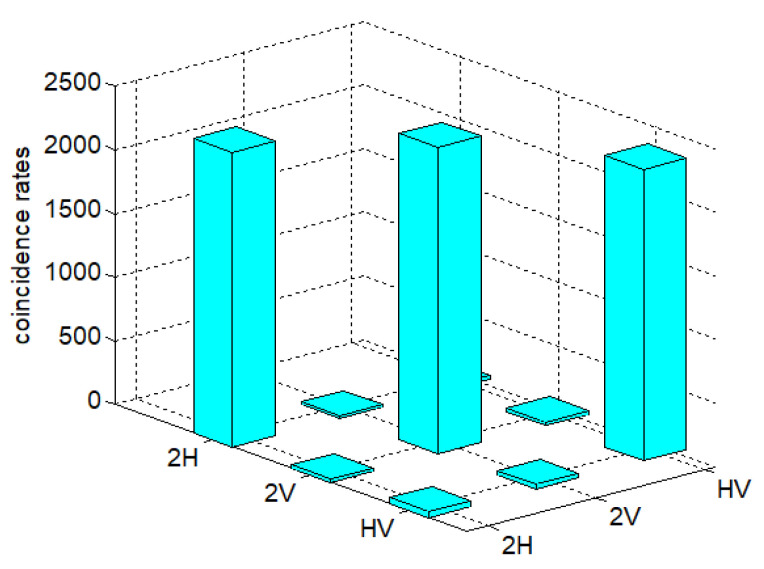
The measurement results of spin-1 states. The data acquisition time is 900 s.

As mentioned above, the experimental verification of the information entropy inequality, especially the high-dimensional information entropy inequality, is very demanding on the fidelity and brightness of the entanglement state. Therefore, we adopted a variety of technologies to improve the fidelity and brightness of the prepared four-photon entangled states: Firstly, we achieved and maintained a constant temperature, constant humidity, and constant mechanical vibration environment for a long time to reduce the fluctuation of laser pumping power. Secondly, we used the optical feedback loop structure to make the ultraviolet pumped light pass through the BBO crystal twice to improve the brightness of the prepared entangled light source [17,18]. Due to the adoption of the feedback loop structure, the count rate of four-photon entangled states was 16 times better and an approximately intensity 7/s was achieved. At the same time, we obtained and used 150 mw as the optimized laser pump power, which can guarantee enough coincidence counts while ensuring high visibility of the quantum state. We then fine-tuned the additional combination of QWP + HWP + QWP to improve the contrast of the entanglement system, as shown in Figure 1. Finally, we got rid of the effect of device loss and dark count on measurement data. We measured the density matrix by the method of over-complete state tomography to characterize the prepared entangled states. The experimental data of nine combinations of measurement basis, |2H〉, |2V〉, and |HV〉, were collected, as shown in Figure 2. Through the above operation, we could obtain the entangled state with a fidelity of 97.8 ± 0.2%, which could prove that the entangled system had good properties [20,21]. 

**Figure 3:**
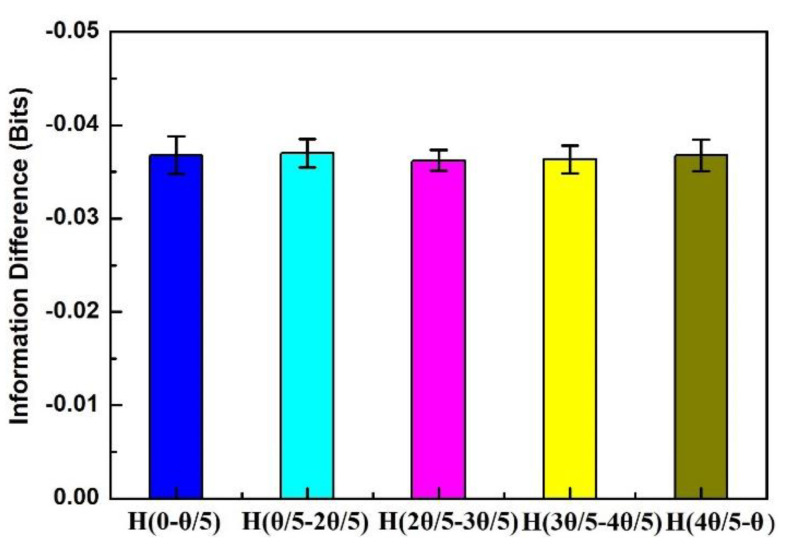
The experimental results of information difference when the *θ* is rotated from 0 to *θ*/5, *θ*/5 to 2*θ*/5, 2*θ*/5 to 3*θ*/5, 3*θ*/5 to 4*θ*/5, and 4*θ*/5 to *θ* within the margin of error. The *θ* value equal to 40° is the angle value generated by the maximum information entropy difference.

The advantage of using information entropy inequality to quantify quantum correlation is that it the amount of experimental measurement observables can be significantly decreased. Thus, according to the symmetry and rotational invariance of the experimental system, we gave the experimental results with information difference of $H{\;}^{\mathrm{QM}}(0,\;\theta /5)$$H{\;}^{\mathrm{QM}}(\theta /5,\;2\theta /5)$, $H^{\mathrm{QM}}(2\theta /5,3\theta /5)$, $H^{\mathrm{QM}}(3\theta /5,4\theta /5)$, and $H^{\mathrm{QM}}(4\theta /5,\theta )\;$ to prove the information entropic inequality. Since we were dealing with widely separated systems, the measurement on particle A did not disturb particle B. This meant that the measurement between any pair was expressed in the form of an appropriate probability. The experimental results and experimental errors of information difference when the *θ* was rotated from 0 to *θ*/5, *θ*/5 to 2*θ*/5, 2*θ*/5 to 3*θ*/5, 3*θ*/5 to 4*θ*/5, and 4*θ*/5 to *θ* are shown in Figure 3. It can be seen that within the error range, the measurement results of these five parts were very close, and the error was likely caused by the modulation of the measurement equipment angle [20].

**Figure 4:**
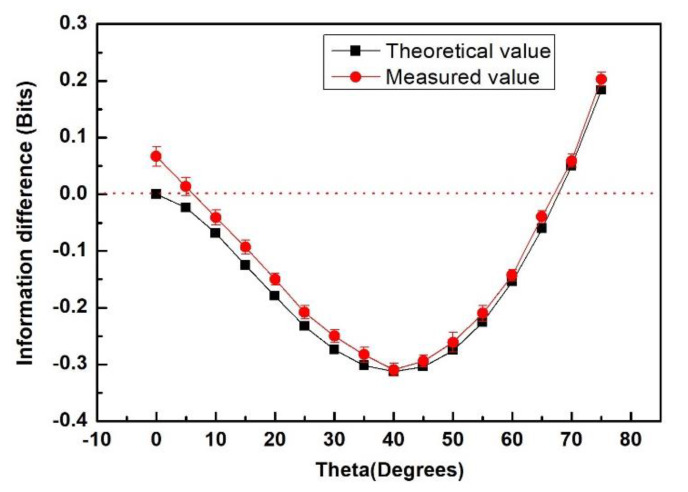
The information differences of information entropic inequality in bits depended on rotation angle θ. The measurement time of each experimental measurement point is 900 s. In order to reduce the measurement error, 50 measurements were made for each measurement point.

The information differences of information entropic inequality in bits dependent on rotation angle *θ*, as shown in Figure 4. Firstly, the theoretical calculation results of spin-1 information entropy inequality are given. Next, we experimentally measured the relationship between information entropy difference and angle *θ*. According to the theoretical prediction, the maximum information deficit is generated when the angle *θ* is 40° and the difference is about 0.32. The experimental value was −0.3122 ± 0.0137. According to Figure 4, we can see that the experimental measurement results agreed well with the theoretical prediction results, except in the small angle area. The significant difference in the small angle region was due to the quantum Zeno effect [4]. At the same time, having a negative value meant there was a quantum correlation and vice versa. It should be pointed out that in Reference [18], the author also proved the experimental violation of a spin-1 CHSH Bell inequality by measuring joint probability using a four-photon entanglement state. In this paper, we verified the information entropy inequality by measuring the mean value of information entropy. This method of measuring information entropy can not only give the contradiction between the local hidden variables theory and quantum mechanics clearly, but can also directly measure the difference of quantum information between two related systems. It is obvious that the measurement of information entropy difference based on information entropy inequality is an effective method to determine high-dimensional quantum correlation.

In the quantification and application of high-dimensional entanglement correlation, there are still many open problems to be studied. Compared to the conventional two-level systems, high dimensional entanglement states can offer extended possibilities, such as higher capacity and noise resilience in quantum communications, quantum simulation, and quantum computation [1,2,3,22,23,24,25]. Therefore, it is of great significance to further expand our work to more than s>1 higher-level entangled systems, especially using the high dimensional information entropic as a tool to study the anti-noise capability and information capacity of high dimensional entanglement systems under different noise environments.

## 5. Conclusions

In this paper, the high quality photonic maximally entangled states were prepared based on the second-order nonlinear optical effect, multiphoton interference enhancement effect, and multiple fidelity optimization techniques. We demonstrated the spin-1 information entropic inequality for the first time using the high quality simulated photonic qutrit states. Our results show that the measurement of information entropy difference based on information entropy inequality is an effective method to determine high-dimensional quantum correlation. At the foundational level, studying the differences gives us insight into the quantum correlation of any pair of widely separated systems, while, on a practical level, these differences are crucial for applications in device-independent quantum information processing schemes.
